# Health literacy and successful aging among elderly patients at Kasr Alainy Hospital: a cross-sectional comparative study

**DOI:** 10.1186/s12889-026-29518-z

**Published:** 2026-10-09

**Authors:** Hend Samy Ibrahim, Eman Ismail Raslan, Eman Hany Elsebaie

**Affiliations:** 1 Department of Public Health and Community Medicine, Faculty of Medicine, Cairo University, Cairo, Egypt; 2 Department of Family Medicine, Faculty of Medicine, Cairo University, Cairo, Egypt

**Keywords:** Successful Aging, Health Literacy, Geriatric Health, Elderly Patients, Healthy Aging

## Abstract

**Background:**

Population aging and chronic disease burden are increasing in Egypt. Health literacy (HL) may play a key role in promoting successful aging (SA), but evidence is limited.

**Aim:**

To assess HL and SA and examine their association among elderly patients at Kasr Alainy Hospital.

**Methods:**

A cross-sectional comparative study of 246 participants (123 patients with chronic diseases and 123 healthy controls) aged ≥ 65 years was conducted using the Health Literacy Questionnaire and Successful Aging Scale.

**Results:**

Patients had significantly lower HL and SA scores than controls (*p* < 0.001). HL was positively correlated with SA (*r* = 0.612, *p* < 0.001) and remained the strongest independent predictor in regression analysis. Higher education and income improved SA, while age, rural residence, and smoking negatively affected it.

**Conclusion:**

Future research should employ longitudinal designs, community-based probability sampling, fully validated Arabic versions of the instruments with reported psychometrics in the study population, and, ultimately, randomized evaluations of health-literacy interventions to establish whether improving HL improves successful-aging outcomes. Health literacy is strongly associated with successful aging among older adults in Egypt. Higher health literacy is associated with better aging outcomes; interventions to improve HL may therefore be beneficial.

## Introduction

A profound global demographic shift is marked by a rapid increase in the elderly population. Projections anticipate that by 2050, approximately 2 billion individuals will be aged 60 and above. This trend is particularly evident in developed nations with robust social safety nets. Still, its impact extends to countries at all stages of development [24].

Egypt, classified as a lower-middle-income country, exemplifies this trend. Currently, around 6.9% of Egypt’s population is aged 65 and above [7]. While this percentage may seem modest compared with some developed nations, it holds significance in Egypt’s context. Moreover, this percentage is expected to be more than double by 2050, reaching around 14% of the total population [25].

These growing elderly population faces a unique set of health challenges. Chronic illnesses like dementia, diabetes, and heart disease are significantly increasing in prevalence as individuals enter their later years. These conditions not only impact the well-being of older adults but also place a significant strain on healthcare systems, particularly in countries with limited resources like Egypt. The growing expenses of managing these chronic illnesses threaten to overwhelm healthcare budgets, necessitating innovative solutions and resource allocation strategies. However, the concept of aging extends beyond the mere absence of disease [22].

The capacity to sustain a high degree of mental and physical function, social interaction, and general well-being in later life is referred to as “successful ageing”. This encompasses factors like maintaining mobility, independence in daily activities, cognitive sharpness, and a sense of purpose and social connection. Interestingly, research suggests that successful aging and an individual’s health literacy are closely related [27].

Health literacy, defined as the capacity to access, comprehend, assess, and utilize health information to make knowledgeable decisions about personal health and healthcare, empowers older adults to actively participate in their own care. Individuals with greater health literacy skills are demonstrably more competent to manage chronic conditions, handle complex healthcare systems, and advocate for their needs. This, in turn, contributes significantly to achieving successful aging outcomes [13, 23].

The relation between health literacy and successful aging is a growing area of research worldwide. However, there’s a scarcity of studies specifically focused on the elderly population within the Egyptian context. This research aims to address this critical gap in knowledge. So, this study aims to assess the health literacy status of elderly patients at Kasr Alainy Hospitals, evaluate successful aging outcomes in this same patient population, and examine the relationship between health literacy and successful aging among these elderly patients.

## Subjects and methods

### Study design and study participants

This study is a cross-sectional comparative study designed to investigate the association between health literacy and successful aging in elderly patients over 65 years. Data collection occurred at the family medicine department of Kasr Alainy Hospital. Although the original protocol used a case-control label, the design is cross-sectional: health literacy and successful-aging scores were measured at the same time point and groups were not individually matched.

#### Patient group

Participants were recruited according to the inclusion criteria, which are:

- Elderly individuals 65 and above, and Willing to participate in the study.
- Diagnosed with at least one chronic illness (such as coronary artery disease, diabetes mellitus, and hypertension).
- [19].

Exclusion criteria include:

- Participants with severe health conditions pose participation risks (e.g., acute coronary syndrome, uncontrolled hemorrhage).
- Participants with cognitive dysfunction (assessed by the attending physician using clinical judgement and, when available, Mini-Mental State Examination score < 24) that could affect questionnaire comprehension were excluded.
- Unwilling to participate.

#### Healthy control group

In addition to the patient group, a healthy control group was recruited. These individuals should:

- Match the age range of the patient group.
- Not having any diagnosed chronic diseases (verified by self-report and review of available medical records and current medications)
- Report generally good health.
- [19].

Like the patient group, cognitive impairment (same criteria) was an exclusion criterion for the healthy controls.

### Sample size and sampling technique

Sample size calculation using EpiInfo 6 software yielded 246 participants (123 patients, 123 healthy controls) with a power of 80%, 95% confidence, and a 5% margin of error. It was calculated based on the prevalence of health literacy among the elderly in a previous study, which was 20.4% [23]. Any patient fulfilling the inclusion criteria was included in the study using the purposive sampling technique.

### Data collection

Following the acquisition of necessary ethical permissions, researchers conducted face-to-face interviews to collect data from participants between October 2024 and March 2025. Of 283 eligible individuals approached, 246 (87%) agreed to participate and completed the questionnaires (response rate 87%). Written informed consent was acquired prior to any data collection. Data were collected between October 2024 and March 2025. Of 283 eligible individuals approached, 246 completed the interviews (response rate 87%). Data were collected between October 2024 and March 2025. Of 283 eligible individuals approached, 246 completed the interviews (response rate 87%).

A standardized questionnaire was used to gather data from participants. The questionnaire is divided into three sections:

#### Section 1: Sociodemographic characteristics and medical history

This section collects information on participants’ age, sex, marital status, education level, prior occupation, family income, and place of residence. Additionally, it gathers details regarding chronic disease diagnoses, disease duration, medication use (including type and quantity), prior hospitalizations, and smoking history (if applicable).

#### Section 2: Health literacy questionnaire (HLQ) [17]

Health literacy in this study goes beyond reading medical jargon. It’s the ability of elderly participants to navigate the complexities of health information: finding reliable sources, understanding medical terms and instructions, using this knowledge to manage their chronic conditions (medication, appointments, symptoms), and communicating effectively with healthcare providers to make sound decisions regarding their health.

The HLQ, a validated and reliable 44-item instrument, was used to evaluate participants’ health literacy in nine domains. Each domain can be analyzed independently, but the sum of the scores provides a total health literacy score. Higher scores are indicative of increased health literacy.

The nine HLQ domains encompass:

- Feeling supported by healthcare providers.
- Having sufficient health information.
- Actively managing personal health.
- Receiving social support for health.
- Appraising health information critically.
- Effectively engaging with healthcare providers.
- Navigating the healthcare system.
- Finding quality health information.
- Understanding health information for decision-making.

Participants utilized a 5-point Likert scale for each item, ranging from (never) (1) to (always) [27]. Total scores will be categorized as follows:

- Poor health literacy: < 50% of total score.
- Fair health literacy: 50% – < 70% of total score.
- Good health literacy: ≥ 70% of total score.

#### Section 3: Successful aging scale (SAS) [19]

Successful aging is a multifaceted concept encompassing a high degree of well-being in three key areas: physical health (maintaining good function, managing chronic conditions, healthy lifestyle choices), mental health (emotional well-being, strong cognitive function, resilience), and social engagement (strong connections, meaningful activities, sense of purpose). These dimensions are interconnected, with physical health influencing mental well-being and social connections providing support for healthy habits.

The SAS scale assesses these dimensions through various aspects. It is a well-established 14-item questionnaire, used to assess participants’ level of successful aging. This scale incorporates both factors contributing to successful aging and the ongoing processes that maintain it. All items contribute to a final overall score, with higher scores indicating a greater degree of successful aging.

The SAS is composed of three subscales:

- Healthy Lifestyle (Items 1, 7, 8, & 13): This subscale assesses participants’ health-promoting behaviors and habits.
- Adaptive Coping (Items 2, 3, 12, & 14): This subscale evaluates participants’ ability to manage stress and challenges effectively.
- Engagement with Life (Items 4, 5, 6, 9, 10, & 11): This subscale measures participants’ level of engagement in activities and social connections that bring meaning and purpose to their lives.

Participants responded to each SAS item using a Likert scale with response options ranging from (strongly disagree) to (strongly agree). The SAS has demonstrated good internal consistency in the original development sample (Cronbach’s alpha = 0.84). In the present sample, internal consistency was excellent for the total SAS (Cronbach’s alpha = 0.87) and good for the HLQ composite score (Cronbach’s alpha = 0.91).

### Statistical analysis

Data analysis was conducted using the statistical package SPSS version 28 (IBM Corp., Armonk, NY, USA). Quantitative data were analyzed using measures of central tendency and dispersion, such as mean and standard deviation, while qualitative data were analyzed using percentages. To compare the patients and control groups, independent sample t-tests and chi-square tests were utilised for quantitative and qualitative variables, respectively. Pearson correlation was used to determine the correlation between HLQ and SAS Scores. Statistical significance was defined as a p-value of less than 0.05.

### Ethical consideration

This study was conducted in strict accordance with the ethical standards and norms established by the Declaration of Helsinki. The study protocol received formal ethical approval from the Faculty of Medicine, Cairo University Research Ethics Committee on October 7, 2024 (approval number N-257–2024).

Every human participant provided their voluntary, written informed consent before any data collection. Participants received explicit information regarding the study’s purpose, procedures, potential risks, and benefits, ensuring voluntary participation without coercion. Confidentiality of personal information was strictly maintained, and all data were anonymized to protect participants’ privacy.

## Results

A total of 246 older adults were included in the study, comprising 123 patients with chronic diseases and 123 healthy controls. The two groups were comparable regarding age, sex, occupation, marital status, residence, and monthly family income, with nostatistically significant differences observed. Educational level differed significantly between the groups (p = 0.014), with a higher proportion of patients having no formal education. The mean age of participants was 72.3 ± 5.3 years in the patient group and 71.6 ± 4.8 years in the control group (*p* = 0.278). However, significant differences were observed in smoking status and prior hospitalization history. Current or former smoking was more prevalent among patients than controls (33.3% vs. 14.6%, *p* < 0.001), while previous hospitalization was reported by more than half of the patient group compared with only 9.8% of controls (54.5% vs. 9.8%, *p* < 0.001) (Table 1).

**Table 1:** Sociodemographic and health characteristics of the participants

Characteristic	Patient Group (*n* = 123)	Control Group (*n* = 123)	*p*-value
Age (years), mean ± SD	72.3 ± 5.3	71.6 ± 4.8	0.278
Sex			0.522
- Male	54 (43.9%)	59 (48.0%)	
- Female	69 (56.1%)	64 (52.0%)	
Education level			0.014
- No formal education	28 (22.8%)	9 (7.3%)	
- Primary	41 (33.3%)	52 (42.3%)	
- Diploma	22 (17.9%)	23 (18.7%)	
- Secondary	14 (11.4%)	21 (17.1%)	
- University	18 (14.6%)	18 (14.6%)	
Occupation			0.057
- Unemployed	18 (14.6%)	12 (9.8%)	
- Self-employed	15 (12.2%)	28 (22.8%)	
- Governmental sector	12 (9.8%)	18 (14.6%)	
- Retired	78 (63.4%)	65 (52.8%)	
Marital status			0.055
- Married	71 (57.7%)	89 (72.4%)	
- Divorced	14 (11.4%)	9 (7.3%)	
- Widow	38 (30.9%)	25 (20.3%)	
Residence			0.305
- Urban	71 (57.7%)	63 (48.7%)	
- Rural	52 (42.3%)	60 (51.3%)	
Monthly family income			0.102
- Less than 5000 EGP/month	48 (39.0%)	54 (43.9%)	
− 5000–10,000 EGP/month	39 (31.7%)	28 (22.8%)	
− 10,000–20,000 EGP/month	24 (19.5%)	35 (28.5%)	
- More than 20,000 EGP/month	12 (9.8%)	6 (4.8%)	
Smoking status			< 0.001
- Yes (current smoker)	22 (17.9%)	8 (6.5%)	
- No (never smoked)	82 (66.7%)	105 (85.4%)	
- Former smoker	19 (15.4%)	10 (8.1%)	
Prior hospitalization			< 0.001
- Yes	67 (54.5%)	12 (9.8%)	
- No	56 (45.5%)	111 (90.2%)	

Among participants in the patient group, hypertension was the most frequently reported chronic condition (84/123, 68.3%), followed by diabetes mellitus (76/123, 61.8%) and coronary artery disease (42/123, 34.1%). Sixty-one patients (49.6%) had two or more of these conditions (multimorbidity). Median disease duration was 8 years (interquartile range 4–12). The median number of regular medications was 4 (range 1–9). No chronic diseases were reported among controls, consistent with the study eligibility criteria and verification procedure.

Health literacy levels differed substantially between groups across all domains of the Health Literacy Questionnaire (HLQ). Patients demonstrated significantly lower scores than healthy controls in every HLQ domain (all *p* < 0.001). The largest between-group differences were observed in the domains related to having sufficient health information, navigating the healthcare system, appraisal of health information, and the ability to find good health information.

**Fig. 1:**
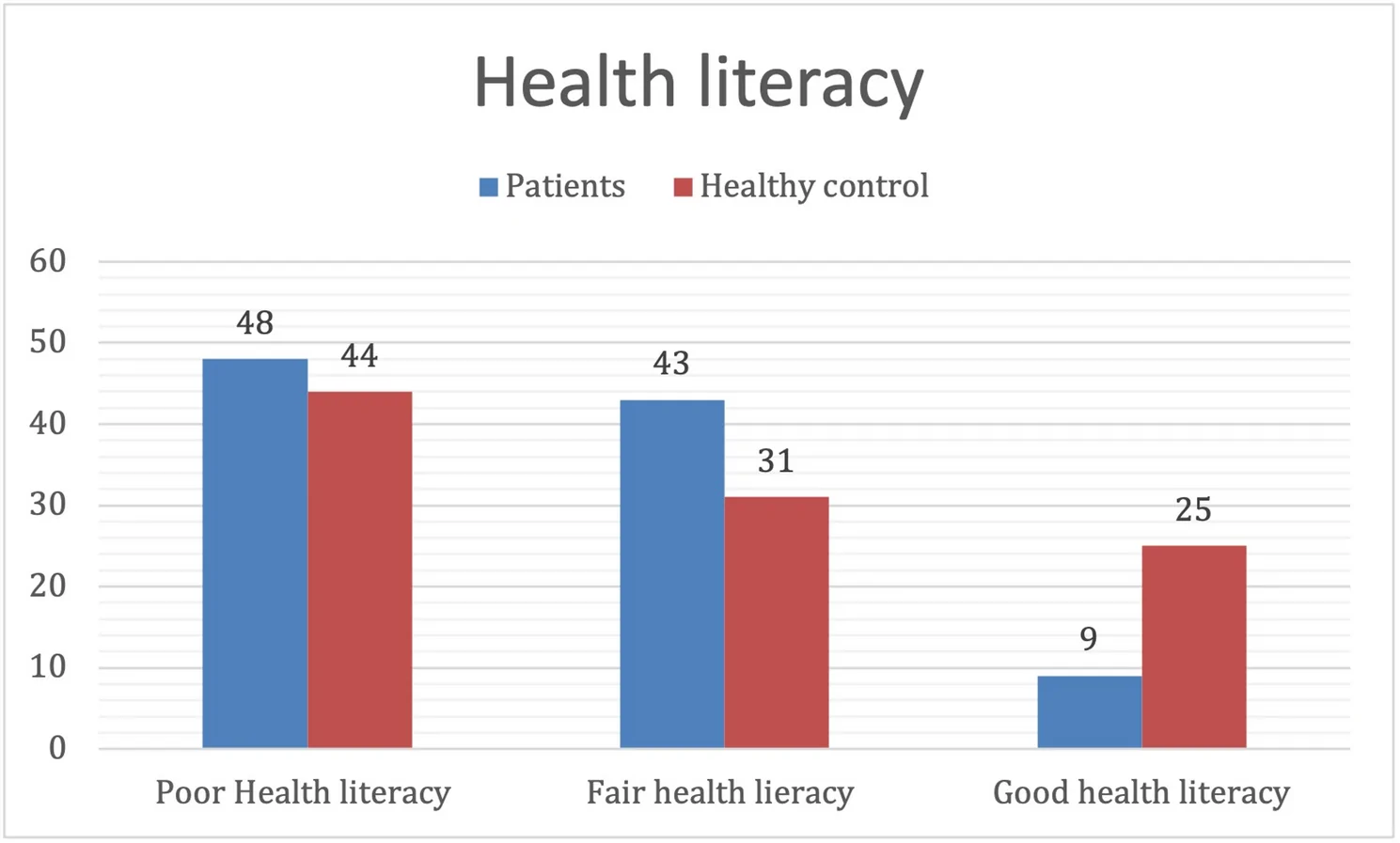
Distribution of health literacy levels among patients and healthy controls. (χ² = 12.046, *p* < 0.001)

The mean total HLQ score was significantly lower among patients than controls (117.8 ± 21.6 vs. 171.5 ± 20.4, *p* < 0.001) (Table 2). Similarly, categorical analysis revealed significantly poorer health literacy among patients (χ² = 12.046, *p* < 0.001). Nearly half of the patient group demonstrated poor health literacy (48%, *n* = 59), compared with 44% (*n* = 54) among controls. In contrast, good health literacy was more common among healthy controls than patients (25%, *n* = 31 vs. 9%, *n* = 11) (Fig. 1).

**Table 2:** Health literacy questionnaire (HLQ) scores

Domain/Scale	Patient Group	Control Group	*p*-value
1. Feeling supported by providers	2.89 ± 0.71	3.78 ± 0.62	< 0.001
2. Having sufficient health information	1.65 ± 0.69	3.85 ± 0.58	< 0.001
3. Actively managing health	2.71 ± 0.74	3.82 ± 0.55	< 0.001
4. Social support for health	2.94 ± 0.82	4.21 ± 0.61	< 0.001
5. Appraisal of health information	2.58 ± 0.72	3.69 ± 0.44	< 0.001
6. Engaging with healthcare providers	3.76 ± 0.68	4.85 ± 0.59	< 0.001
7. Navigating the healthcare system	2.41 ± 0.68	3.72 ± 0.63	< 0.001
8. Ability to find good health information	2.67 ± 0.65	3.81 ± 0.57	< 0.001
9. Understanding health information	2.82 ± 0.70	3.88 ± 0.60	< 0.001
Composite HLQ score (sum of domain means)	117.8 ± 21.6	171.5 ± 20.4	< 0.001

Successful aging scores were significantly lower among patients with chronic diseases compared with healthy controls across all domains of the Successful Aging Scale (SAS). The mean total SAS score was 40.8 ± 4.9 in the patient group compared with 55.2 ± 4.1 among controls (*p* < 0.001) (Table 3).

**Table 3:** Successful aging scale (SAS) scores

Subscale/Scale	Patient Group	Control Group	*p*-value
Healthy Lifestyle (4 items)	11.2 ± 2.1	16.4 ± 1.8	< 0.001
Adaptive Coping (4 items)	12.1 ± 2.3	15.8 ± 1.9	< 0.001
Engagement with Life (6 items)	17.5 ± 3.1	23.0 ± 2.7	< 0.001
Total score (out of 70)	40.8 ± 4.9	55.2 ± 4.1	< 0.001

Significant between-group differences were also observed across all SAS subscales, including healthy lifestyle, adaptive coping, and engagement with life (all *p* < 0.001). The greatest disparities were identified in the healthy lifestyle and engagement with life domains.

Pearson correlation analysis demonstrated significant positive associations between health literacy and successful aging outcomes. Total HLQ score showed a strong positive correlation with total SAS score in the overall sample (*r* = 0.612, *p* < 0.001). Positive correlations were consistently observed between all HLQ domains and SAS subscales, with correlation coefficients ranging from 0.44 to 0.68 (all *p* < 0.01) (Table 4).

**Table 4:** Pearson correlation coefficients between HLQ and SAS scores

HLQ Domain/Scale	Healthy Lifestyle	Adaptive Coping	Engagement with Life	Total SAS Score	*P* value
Feeling supported by providers	0.48	0.45	0.51	0.53	< 0.01
Having sufficient health information	0.55	0.47	0.52	0.57	< 0.01
Actively managing health	0.68	0.52	0.49	0.62	< 0.01
Social support for health	0.46	0.51	0.58	0.56	< 0.01
Appraisal of health information	0.53	0.49	0.47	0.54	< 0.01
Engaging with healthcare providers	0.51	0.44	0.55	0.55	< 0.01
Navigating the healthcare system	0.65	0.50	0.48	0.60	< 0.01
Ability to find good health information	0.57	0.46	0.50	0.56	< 0.01
Understanding health information	0.54	0.48	0.53	0.58	< 0.01
Total HLQ Score	0.64	0.55	0.58	0.612	< 0.01

Subgroup analyses demonstrated that the association between health literacy and successful aging remained significant within both study groups, although the relationship was stronger among patients with chronic disease (*r* = 0.589, *p* < 0.001) than among healthy controls (*r* = 0.478, *p* < 0.001).

Multiple linear regression analysis was performed to identify independent predictors of successful aging using the total SAS score as the dependent variable. After adjustment for sociodemographic and behavioral variables, total HLQ score remained the strongest independent predictor of successful aging (standardized β = 0.481, *p* < 0.001).

Higher educational level and higher monthly family income were independently associated with higher SAS scores (β = 0.218, *p* < 0.001, and β = 0.167, *p* = 0.001, respectively). In contrast, increasing age (β = −0.112, *p* = 0.020), rural residence (β = −0.134, *p* = 0.004), and current or former smoking (β = −0.109, *p* = 0.017) were independently associated with lower successful aging scores (Table 5).

**Table 5:** Multiple linear regression analysis: predictors of successful aging (SAS total score)

Predictor Variable	Unstandardized β (B)	Standardized β	t-value	*p*-value
Total HLQ score	0.142	0.481	9.87	< 0.001
Age (years)	−0.089	−0.112	−2.34	0.020
Education level (higher)	1.245	0.218	4.12	< 0.001
Monthly family income (higher)	0.876	0.167	3.45	0.001
Rural residence (yes vs. no)	−2.341	−0.134	−2.89	0.004
Current or former smoking (yes vs. no)	−1.678	−0.109	−2.41	0.017

The overall regression model was statistically significant (F = 28.76, *p* < 0.001) and explained 45.3% of the variance in successful aging outcomes (adjusted R² = 0.453). 95% confidence intervals for the standardized coefficients were: total HLQ β = 0.481 (95% CI 0.385–0.577), age β = −0.112 (95% CI − 0.206 to − 0.018), education β = 0.218 (95% CI 0.114–0.322), income β = 0.167 (95% CI 0.072–0.262), rural residence β = −0.134 (95% CI − 0.225 to − 0.043), smoking β = −0.109 (95% CI − 0.198 to − 0.020). All VIFs were < 2.5.

## Discussion

The present study holds considerable importance as one of the first cross-sectional comparative investigations in Egypt to directly examine the relationship between health literacy (HL) and successful aging (SA) among older adults with chronic conditions compared to healthy controls. By demonstrating that HL remained the strongest independent associate of SA after adjustment for key sociodemographic and behavioral factors, the study addresses a critical evidence gap in a middle-income setting where population aging is accelerating rapidly—from 8.8% of Egyptians currently aged 60+ (approximately 9.3 million) to a projected 17.9% by 2052 [26]. This local evidence is particularly valuable because it highlights HL as a modifiable factor associated with empowerment of older adults with multimorbidity (e.g., hypertension, diabetes, and coronary artery disease) to achieve better self-management, lifestyle choices, and life engagement.

The disparity in health literacy levels observed in Fig. 1—where poor and fair health literacy are substantially more prevalent among patients than healthy controls, while good health literacy is considerably higher among controls (*p* < 0.001)—highlights a significant association between chronic disease status and lower health literacy in this population. This finding suggests that individuals with chronic conditions may face greater challenges in accessing, understanding, and utilizing health information necessary for effective disease management. Limited health literacy among patients with chronic diseases may contribute to difficulties in adhering to treatment recommendations, navigating healthcare services, and adopting appropriate self-care practices [9]. This high prevalence of poor HL among patients aligns closely with emerging evidence from Egypt and the broader Arab region, where inadequate health literacy is alarmingly common among elderly people, particularly those with chronic illnesses [12]. For example, a recent assessment of diabetes-specific health literacy among diabetic elderly individuals visiting geriatric clubs in Egypt reported inadequate levels in 99.2% of participants, accompanied by low diabetes awareness and subpar self-care habits [29].

Similarly, in Alexandria, Egypt, elderly people with type 2 diabetes showed varying but often limited HL capabilities that negatively influenced self-care activities, with HL and diabetes knowledge emerging as significant indicators of better self-care [2]. Broader regional patterns reinforce these deficits: studies in the United Arab Emirates have reported inadequate HL in over 60% of surveyed populations [16], while in Saudi Arabia, low or limited HL has been documented in approximately 46% of adults, with higher burdens among those with chronic conditions [4]. Additionally, among caregivers of elderly patients in Egypt, only 25% exhibited adequate HL, with low levels linked to increased hospitalization frequency, prolonged hospital stays, and lower health-related quality of life for the elderly [18]. These consistent findings indicate that low HL is a widespread systemic barrier in middle-income Arab contexts, exacerbating multimorbidity burdens, hindering effective chronic disease management, and contributing to poorer aging outcomes—precisely as evidenced in our patient group, where poor HL predominated and strongly correlated with reduced SA scores.

These findings align with other studies from different countries. Kulakçı-Altıntaş and Ayaz-Alkaya, [11] reported that HL and SA are mutually predictive in older Turkish adults, with a model incorporating age and health perception explaining 22% of variance in SA scores. The stronger explanatory power observed here (adjusted R² = 0.453) likely stems from the inclusion of context-specific covariates (rural residence, income, smoking) that amplify the impact of HL in resource-constrained environments.

Within the Arab region and Egypt specifically, emerging studies support the centrality of HL in aging outcomes. Arafat et al., [6] reported a statistically significant positive correlation between health behaviors and self-perception of SA in a rural Egyptian sample (Sharkia governorate), where 50.0% exhibited moderate SA levels. Shaltout, [21] emphasized diet, lifestyle, and social relations as determinants of SA in Egypt, noting that improved HL could enhance these factors. Regionally, Alhazmi et al., [3] linked sustainable healthy eating behaviors to higher SA scores among community-dwelling older adults in Abha, Saudi Arabia.

Some contradictory findings appear in the broader literature. Certain international studies in high-resource settings report weaker associations or lower explained variance between HL and SA compared with the present results [10]. This discrepancy can be attributed to differences in healthcare infrastructure: in well-resourced systems (often high-income countries), external support—such as comprehensive patient navigation programs, universal health coverage, and robust primary care—may buffer the consequences of limited individual HL, reducing its relative predictive strength on health outcomes and aging trajectories [1, 20]. In contrast, in Egypt and similar Arab middle-income contexts—where structural barriers such as rural–urban disparities, variable healthcare access, high multimorbidity burden, and underdeveloped social protection systems prevail—HL assumes greater importance as a personal resource for navigating fragmented systems and managing chronic conditions effectively [5, 14, 15]. These contextual differences amplify HL’s role in LMICs, where low HL exacerbates inequities in chronic disease self-management and access to preventive services, leading to poorer functional outcomes in older adults.

The mechanistic pathways suggested by the domain-specific associations (particularly in information sufficiency, healthcare navigation, and appraisal) align with established frameworks: adequate HL enables older adults to obtain, understand, and apply health information, thereby supporting informed lifestyle decisions, adaptive coping, and active engagement—core elements of SA [8]. Sociodemographic patterns observed (positive effects of education and income; negative effects of rural residence, smoking, and age) further reflect documented social determinants of healthy aging in low- and middle-income countries.

The practical value of this study lies in its provision of actionable, locally generated evidence. It demonstrates that HL is not merely a correlate, but a modifiable factor associated with successful aging even among older adults already living with chronic disease. By bridging the gap between health information and effective action, targeted HL enhancement may be associated with improved chronic-disease outcomes, lower hospitalization rates, and better quality of life—benefits that are especially timely given Egypt’s demographic transition and the rising burden of non-communicable diseases.

From a policy perspective, these results carry direct implications for geriatric care in Egypt and the broader Arab region. National strategies for aging, such as those aligned with the WHO Decade of Healthy Ageing and Egypt’s evolving elderly care frameworks, including the National Health Strategy 2024–2030 [28], might integrate routine HL screening into primary and geriatric services. Culturally tailored, low-literacy interventions—simplified patient education materials, community-based navigation programs, and accessible digital tools—represent cost-effective approaches that can simultaneously strengthen chronic-disease management and promote successful aging. Such measures would help alleviate pressure on overstretched healthcare systems and support older adults in aging with greater functional independence and functional independence.

## Conclusion

Several limitations should be noted. First, in the original protocol group membership was partly defined by a Successful Aging Scale threshold. This created circularity when SAS scores were subsequently compared and modelled as the outcome. In the revised analysis, groups are defined exclusively by the presence or absence of chronic disease, and SAS is analyzed only as a continuous outcome. Second, participants were recruited by purposive sampling from the family-medicine outpatient department of a single tertiary hospital (Kasr Alainy). Attendees of this setting may differ systematically from community-dwelling older adults; findings should therefore be generalized only to similar clinical populations. Third, the design is cross-sectional, so causal direction cannot be established. Finally, the HLQ composite score used in regression requires careful interpretation because the nine domains employ different response scales. In addition, the categorical distribution of health-literacy levels (Fig. 1) and certain domain means require verification against the raw dataset to ensure consistency with the reported means and sample sizes. The original sample-size calculation was based on a prevalence estimate rather than an expected between-group difference, which is suboptimal for a comparative design. Health literacy is strongly associated with successful aging among older adults in Egypt, especially those with chronic conditions. Patients with chronic diseases exhibited lower HL than healthy controls, reflecting challenges in managing health effectively. As a modifiable factor, HL is associated with greater empowerment of older adults to improve self-care, lifestyle behaviors, and engagement in life. Integrating culturally tailored HL interventions into geriatric and primary care services can enhance aging outcomes, reduce hospitalizations, and support functional independence in Egypt’s rapidly growing elderly population.

## Data Availability

The datasets used and analysed during the current study are available from the corresponding author on reasonable request.
